# A Retrospective Study of Maternal and Neonatal Outcome in Placenta Accreta Spectrum After Planned or Emergency Delivery From a Tertiary Care Centre in North India

**DOI:** 10.7759/cureus.44725

**Published:** 2023-09-05

**Authors:** Ruchi Birendra, Singh Jigyasa

**Affiliations:** 1 Obstetrics and Gynaecology, Institute Of Medical Sciences, Banaras Hindu University, Varanasi, IND

**Keywords:** placenta accreta spectrum disorder, cesarean hysterectomy, ultrasonography (usg), previous cesarean section, massive hemorrhage

## Abstract

Aim

This retrospective study aimed to evaluate the incidence, sociodemographic profile, feto-maternal outcomes, and associated risk factors of placenta accreta spectrum (PAS) among all the deliveries.

Methods

This retrospective cohort study included all women diagnosed with PAS either preoperatively or intraoperatively. Data on maternal high-risk factors such as previous surgical history, association with placenta praevia, parity and primary outcomes such as the operative procedure carried out, transfusion requirements and ICU admission, as well as neonatal variables such as Apgar score, NICU admission and birthweight, were among the primary outcomes of this study. The study was carried out over a period of 10 months at our centre.

Results

A cohort of 32 women were identified with placenta accreta, increta, or percreta. The mean maternal age was 31 years. The mean gestational age at the time of diagnosis was 32.75 weeks. Around 50% of patients had risk factors for the abnormally invasive placenta, such as placenta praevia, and 75% had a history of previous caesarean sections. Hysterectomy was done in 28 cases (87.5%). Blood transfusion was done in all the cases. There were two maternal deaths in the study group. The perinatal outcome was better in the antenatally detected cases.

Conclusion

An increased incidence of PAS has been seen. Early risk factor identification and strategic management improve maternal and foetal outcomes. Our findings demonstrated that PAS pregnancies managed in our centre had maternal and neonatal outcomes comparable to those in developed countries. It is hypothesized that this is because pregnancies with PAS are managed using a multidisciplinary approach.

## Introduction

Placenta accreta spectrum (PAS) is a disorder of abnormal placentation. PAS is a major obstetrical problem. An increase in the incidence of PAS has been seen with the increase in caesarean deliveries in the last four decades [1]. The other risk factors for PAS include other uterine surgery, including repeated endometrial curettage or caesarean or a history of accreta in a previous pregnancy [2], previous history of retained placenta, and advanced maternal age [3]. Ultrasound, when performed by a skilled operator, is accurate in diagnosing PAS [2]. Currently, there is no evidence of an effective biomarker for serological screening of PAS [2,3].

The most common reason for maternal admission in cases of PAS is vaginal bleeding. The second-most common reason for admission is the antenatal diagnosis of PAS. There is no consensus for a single approach for the management of PAS disorders, and optimal management depends on local expertise. PAS is associated with increased maternal and neonatal morbidity and mortality which can be reduced if these cases are managed by a multidisciplinary team with immediate access to blood products and ICU facilities [2]. These disorders are linked to severe maternal and neonatal morbidity and mortality, even in developed nations with easily available knowledge and cutting-edge resuscitative and supporting facilities [4]. Thus, prenatal diagnosis and management in a tertiary centre are essential to ensure better outcomes. Caesarean hysterectomy after delivery of the baby with placenta in situ is the most widely acknowledged treatment for PAS as attempts at its removal are associated with a considerable risk of haemorrhage [1]. It is linked with much lower morbidity - 36% vs. 67% - compared to those with attempted manual placental removal, therefore antenatal diagnosis is also crucial in these cases [5].

Methotrexate and arterial embolization are not recommended currently since studies have not demonstrated their effectiveness in lowering these risks [6]. Recently, the practice of inserting inflatable balloons into the pelvic arteries, typically in the anterior divisions of the internal iliac arteries, gained popularity. A consistent decrease in surgical blood loss as a result of the incremental improvements in surgical expertise is highlighted in an author’s manuscript [7].

Planned delivery from 35+0 to 36+6 weeks of gestation, in the absence of other risk factors provides the optimal balance between maternal and neonatal health [2,5]. Placenta accreta rates are thought to have grown dramatically during the 1950s. Recent estimates suggest the incidence at 1 in 533 to 1 in 2510 deliveries, compared to rates of 1 in 30,000 births in the 1930s and 1950s. Estimates of the incidence of placenta accreta vary greatly due to differences in diagnostic criteria and study population; nonetheless, there is clear evidence in the literature that the rate has grown significantly over time [8]. The incidence of PAS ranged from 0.01 to 1.1%, according to a recent meta-analysis of population-based research, with a combined prevalence of 0.17% [9].

Studies have used various diagnostic criteria over time, with a contrast between those that accept both clinical and pathologic evidence and those that rely solely on histopathologic diagnosis, typically from hysterectomy specimens, which is one of the reasons why estimates of the incidence of placenta accreta vary so widely [8].

With a focus on demographic details, diagnosis, and clinical treatment outcomes, the objective of this retrospective study was to analyze the outcomes of patients with PAS disorders treated at our tertiary care facility over a period of 10 months.

## Materials and methods

Source of data

Over a 10-month period starting in January 2022 and up to in October 2022, we conducted a retrospective cohort analysis of pregnant patients diagnosed with PAS and placenta previa and treated at our institution. Data was collected from the departmental records and registry system of the obstetric department. This data formed the basis for analysis in the study.

Prior to the start of the study, approval had been acquired by the Institutional Ethical Committee (IEC/Dean/2022/EC 4042; dated 15/04/2023, IMS BHU Varanasi). Each patient's medical record was examined to ascertain demographic characteristics, including age, socioeconomic status, place of residence, parity, their obstetric history, previous exposure to uterine surgery as well as maternal and perinatal treatment and pregnancy outcomes.

Inclusion criteria

All patients identified as having placenta accreta, increta, or percreta by USG or MRI scans and all patients who were not diagnosed preoperatively but whose placenta was found adherent intraoperatively and required active management were included in the study.

Exclusion criteria

All antenatal patients without placenta accreta spectrum were excluded from the study.

Preoperative evaluation

Preoperative diagnosis, requirement for transfusions, length of hospital stay, admission to the ICU, complications following surgery, and neonatal outcome, such as baby weight, APGAR score and need for NICU admission have all been recorded. For cases that were prenatally diagnosed, a senior obstetrician and a consultant anaesthetist carried out the procedure with the assistance of a consultant paediatrician. Blood products were arranged preoperatively in diagnosed cases. Antenatal steroids were given to all patients for maturation of the foetus's lungs after 26 weeks. For this purpose, dexamethasone injection was used. The placental incision was avoided during the operation, which was determined through preoperative ultrasound evaluation. The baby was born via circumnavigating the placenta, and the senior paediatrician immediately received the baby for immediate neonatal care.

Statistical analysis

The data were presented using frequencies, percentages for categorical variables, means, and standard deviations for continuous variables using basic descriptive statistics. All data analysis was performed using IBM SPSS Statistics for Windows, Version 24.0 (IBM Corp., Armonk, NY).

## Results

Sociodemographic characteristics of the patients are depicted in Table 1.

**Table 1:** Demographic characteristics of the study population with placenta accreta spectrum (PAS).

S.N.	Variables	Range		Frequency	Percentage (%)
1	Maternal age (weeks)	>30		11	34.30
		≤30		21	65.70
2	Area of residence	Rural		10	31.25
		Urban		22	68.75
3	Parity	2		8	25
		≥3		24	75
4	History of previous surgical intervention	Dilation and curettage		4	12.5
		Myomectomy		2	6.25
		Caesarean section	1	9	28.13
			2	11	34.30
			≥3	4	12.50
5	Gestational age (weeks)	<30		5	15.63
		≥30		27	84.38

The mean maternal age was 31 years. The mean gestational age at the time of diagnosis was 32.75 weeks. There were 32 cases of PAS during the specified time interval which were diagnosed by histopathology. The total number of deliveries was 2985 out of which 1175 were normal vaginal deliveries and 1810 were deliveries via caesarean section. The calculated incidence of PAS amongst our study group was 10.72 per 1000 deliveries.

There were 152 cases of antepartum haemorrhage (APH) during the specified period. Out of 152 cases of APH, 92 cases were of placenta praevia, 54 were of abruptio placentae and in the remaining six cases, the cause was not known. Thus, the calculated incidence of PAS amongst patients presenting with APH is 21%. Placenta previa was present in 16 cases of PAS. The calculated incidence of placenta previa in PAS was 50%. Eleven (34.37%) patients of PAS with placenta previa had a history of a caesarean section in a previous pregnancy.

There were two cases of PAS in multiparous women with previous normal delivery and in whom there was no known exposure to uterine surgery. Four cases occurred in patients with a prior history of dilation and curettage (D&C) and in two cases there was a history of myomectomy. PAS was diagnosed in 24 patients who had a prior history of caesarean section (75%). Thus, prior caesarean section is shown to be an important risk factor for PAS.

Sixteen (50%) of these women were diagnosed by ultrasound and magnetic resonance imaging (MRI), and 16 (50%) by ultrasound only. There were four cases of APH that the USG interpreted as PAS, although there was no evidence of placental adhesion during surgery.

The most common reason for maternal admission to the hospital was vaginal bleeding which was present in 24 cases. Antenatal diagnosis was done in 18 (56%) of cases. There were cases in which an antenatal diagnosis was made, however, the patient presented to us after the bleeding started. Eight patients presented as placenta previa but were diagnosed as PAS after an MRI was done.

Maternal outcome

Hysterectomy was done in 28 cases. Four cases were managed by a conservative approach due to focal adherent placenta and the bleeding was controlled after surgical management with step-wise devascularisation. Internal iliac artery ligation was done in four cases. There was one case in which the patient had her delivery elsewhere and was referred for retained placenta. PAS was diagnosed by USG and we proceeded with a caesarean hysterectomy. The mean postoperative hospital stay was 10 ± 4 days.

There were 37 cases of maternal deaths that occurred during this time period in our institute. Thus, the calculated maternal mortality ratio (MMR) during this time period is 1239.5 per 100,000 live births. Out of 37 deaths, two deaths were due to PAS. Massive haemorrhage caused irreversible haemorrhagic shock which resulted in multiple organ dysfunction syndrome (MODS) and disseminated intravascular coagulation (DIC) in these patients.

There was one case in which the patient was referred to our centre with retained placenta and PPH. USG was done to confirm PAS. A hysterectomy was conducted for this patient immediately after the diagnosis, but the patient expired later as she had gone into irreversible haemorrhagic shock.

In another case, the morbidly adherent placenta was diagnosed intraoperatively. There was also involvement of the posterior bladder wall. Total abdominal hysterectomy was decided upon due to uncontrollable placenta bed bleeding. A urologist was also called to repair the bladder. After achieving haemostasis, an abdominal drain was kept in place. During surgery, she received four units of whole blood transfusion. She underwent a second surgery three hours after the first one because she had considerable intra-abdominal bleeding. On re-exploration, there was about one litre of hemoperitoneum and multiple oozing from the posterior bladder surface. Evident bleeding spots were located, ligated, and BIIAL (bilateral internal iliac artery ligation) was performed. This patient later succumbed to disseminated intravascular coagulation (DIC) and multiple organ dysfunction syndrome (MODS).

Intraoperative and postoperative data of the study participants are depicted in Table 2.

**Table 2:** Intraoperative and postoperative data of the study participants. CS: caesarean section, DIC: disseminated intravascular coagulation, ICU: intensive care unit, PAS: placenta accreta spectrum disorder

S.N.	Variables	Range	Frequency	Percentage (%)
1	Placenta previa	Yes	16	50
		No	16	50
2	Mode of delivery	CS with placental removal	4	12.5
		CS with placenta left in situ followed by Caesarean hysterectomy	28	87.5
3	Need of blood transfusion		32	100
4	Length of hospital stay (days)	<7	5	15.64
		≥7	27	84.36
5	Intraoperative and postoperative complications	Bladder injury	6	18.75
		DIC	4	12.5
6	Need of admission in ICU		22	68.75
7	Duration of surgery (hours)	<1	4	12.5
		1-2	8	25
		≥2	20	62.5
8	Reasons of admission	Vaginal bleeding	24	75
		Antenatal diagnosis of PAS	18	56.25
		Labour pain	4	12.5
		Elective caesarean	10	31.25
		Placenta previa	8	25

**Figure 1:**
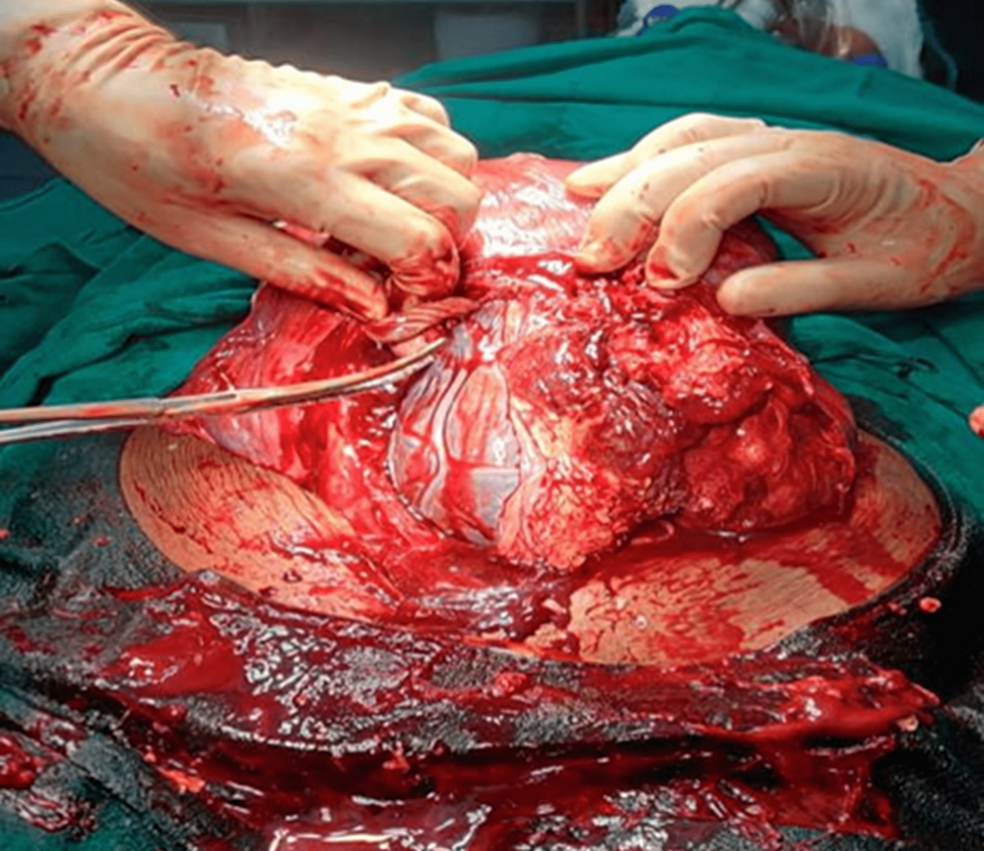
Intraoperative picture of placenta accreta spectrum (PAS) disorder with placenta in situ and proceeding with total hysterectomy.

Figure 1 shows an intraoperative picture of a 28-year-old patient with PAS with placenta in situ undergoing a total hysterectomy.

**Figure 2:**
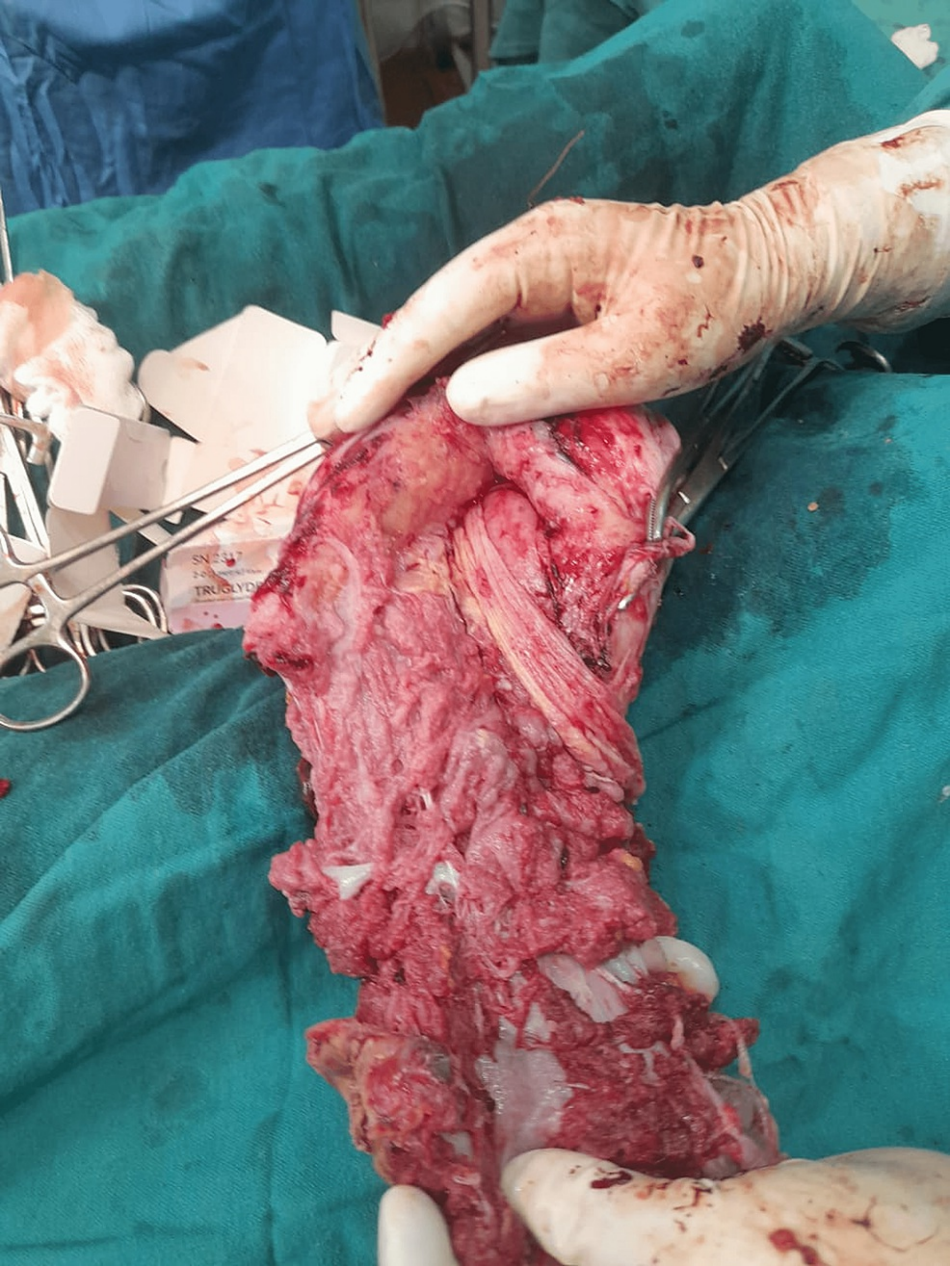
Hysterectomy specimen with placenta in situ of a 27-year-old patient with placenta accreta spectrum (PAS).

Figure 2 shows the hysterectomy specimen with placenta in situ of a 27-year-old patient with PAS.

Neonatal outcome

Most of the pregnancies terminated at a mean gestational age of 32 weeks. Foetal Apgar scores were good in the group of patients who had elective caesarean delivery and were unaffected by PAS risk factors. The patient who had an emergency caesarean section, on the other hand, had a lower foetal Apgar score. After delivery, 14 infants required admission to the neonatal ICU. The most frequent reason for newborn ICU admission was prematurity. There were four cases of neonatal deaths in the emergency caesarean section groups. The level of placental invasion did not significantly alter the foetal Apgar scores, although the gestational age at delivery did.

Neonatal outcomes were summarized in Table 3.

**Table 3:** Table showing neonatal outcomes

S.N.	Variables	In planned LSCS, N (%)	In emergency LSCS, N (%)
1	Apgar score below 7 at five minutes	2 (6.25)	14 (43.75)
2	NICU admission, n (%)	2 (6.25)	12 (37.5)
3	Neonatal death	0	4 (12.5)

## Discussion

The incidence of PAS in our institute is 10.72 per thousand deliveries which is much higher as compared with the one study which reports the incidence of PAS in one centre as 2.3 cases per 1000 deliveries [10]. The incidence of PAS at our hospital is increased perhaps due to its status as a tertiary referral centre. One explanation for this would be the rising prevalence of PAS diseases being treated in specialized facilities, as advised by the ACOG (American College of Obstetricians and Gynaecologists) guidelines.

There is also a wide variation in the incidence of PAS reported in various studies worldwide. For instance, a case-control study in the United States, that examined 64,359 deliveries between 1982 and 2002 found that the prevalence of placenta accreta was 1 in 533 (0.19%) and that it rose in tandem with the rising caesarean rate [11]. These variations may be caused by a variety of reasons. One of the reasons is that there is no universally accepted clinical definition for these conditions. The gold standard for diagnosis of placenta accreta, increta, and percreta is pathological diagnosis, which is obviously only applicable in situations where hysterectomy has been done [12].

PAS was associated with a history of previous caesarean section in 75% of cases in our study. Around 18.75% of patients had a history of D&C and myomectomy. Although only 19 of the total 52 pregnant women with a history of caesarean sections had placenta accreta, the link between the two was shown to be highly statistically significant in one study [13]. Placenta accreta/ increta/ percreta risk was also higher in women who had previous uterine surgery, such as myomectomy [12,13]. The placenta accreta was found in 0.24% of the population at the first caesarean, 0.31% at the second, 0.57% at the third, 2.13% at the fourth, 2.33% at the fifth, and reached values of 6.74% in the sixth caesarean section, according to a cohort study of repeated caesarean deliveries (with 83,754 caesarean deliveries), published by Silver et al. in 2006 [14].

PAS was present in 11 patients who had a history of previous caesarean section along with a low-lying placenta in the present pregnancy. This comprises 34.375% of the total PAS cases. Women who have had a placenta previa and one or more repeat caesarean deliveries are more likely to develop PAS. Because of this, it is crucial to emphasize a diagnostic suspicion when a placenta previa coexists with the history of a caesarean section. According to Silver et al., the risk in these women is 40% or more after a third caesarean [14]. Patients having a history of caesarean section and placenta previa in the present pregnancy accounted for one-third of these PAS cases in a study published by Fitzpatrick et al. [15], which is the same as in our study.

PAS was associated with placenta previa in 50% of cases in our study. A similar association was seen in one study in which out of 198 individuals, 127 (64%) were also diagnosed with placenta previa [3].

There were two cases of PAS diagnosed in multiparous women with no previous surgical history. Similarly, there were five cases in one study that had no identifiable risk factors of PAS. This finding requires physicians to evaluate the patients’ individualized risk factors for developing PAS in the current pregnancy [3].

There were four cases of APH that were diagnosed as PAS by USG but intraoperatively no adhesion of the placenta was seen. This may be due to the fact that the ultrasonography in our institute was done by junior residents, in case of emergency and the residents lacked the expertise to diagnose such situations. USG, when carried out by a trained professional with knowledge in diagnosing PAS is very accurate in diagnosing PAS [2]. The overall diagnostic accuracy of ultrasound in specialized centres is 90.9%, according to a recent systematic review and meta-analysis of prenatal ultrasound diagnosis of placenta previa with PAS in women with a history of cesarean delivery [9].

The overall diagnostic accuracy of ultrasound in specialized centres is 90.9%, according to a recent systematic review and meta-analysis of prenatal ultrasound diagnosis of placenta previa with PAS in women with a history of cesarean delivery [9]. Similarly, there were four cases of PAS which were not diagnosed by ultrasonography in our study. Similarly in a UK-based descriptive study, one of the 20 unsuspected patients with placenta praevia and a prior caesarean section was found to have no ultrasound signs of a morbidly attached placenta [15]. The range of ultrasonography's positive predictive value for placenta accreta is between 65% and 93% [6]. For this reason, it is advised to postpone any surgical intervention until the placenta spontaneously separates after the baby is delivered. Clinically, the absence of placenta separation supports placenta accreta. The sensitivity of ultrasound testing has grown to about 90% because of the introduction of Doppler ultrasound markers. The rate of prenatal detection is affected by the ultrasound signs employed, the operator's experience, the scanning settings, the equipment used, and the gestational age [9].

Hysterectomy was performed in 87.5% of cases in our study. Similarly, hysterectomy was performed in 91.6% of cases in a study from a tertiary centre [6]. Conservative or expectant management should be considered only for carefully selected patients with PAS after thorough counselling regarding the dangers, unknown benefits, and efficacy [16]. Similarly, in accordance with hospital protocol, a caesarean hysterectomy was performed on all patients with PAS in one of the studies [3]. The RCOG (Royal College of Obstetricians and Gynaecologists) and ACOG protocols advocate for careful blood loss monitoring, correction of coagulation problems, and treatment of hydro-electrolytic diseases. The patient's surgical care in the intensive care unit continues to correct vital indicators [6]. In another study, a planned caesarean hysterectomy was performed in two-thirds (67%) of prenatally identified PAS cases, which is the preferred therapeutic strategy for known PAS, according to expert opinion [17]. In known PAS, intentional vaginal delivery is not a frequently acknowledged choice.

Palacios Jaraquemada et al. demonstrated in a multi-centre retrospective case series of 452 patients with invasive placenta that the uterus can be retained with minimum morbidity and blood loss when employing the resection-reconstruction strategy in nearly 80% of instances [18]. Subsequent pregnancies after conservative treatment for PAS (resection-reconstruction) had identical perinatal outcomes to caesarean delivery, with no variations in pregnancy course.

A multidisciplinary approach to PAS management is also vital. A prior study found that as compared to the non-multidisciplinary group, pregnancies managed by a multidisciplinary approach experienced less estimated blood loss, with a trend toward fewer blood transfusions, and were less likely to be delivered emergently [19]. As a result, employing a multidisciplinary approach is one of the possible reasons why our patients' outcomes were comparable to those in developed healthcare settings [3].

Preterm birth, low birth weight, and poor 5-min Apgar scores were among the most frequent unfavourable outcomes for neonates, according to a comprehensive evaluation of 34 studies, which was also found in our study [20].

## Conclusions

Primary prevention requires understanding modifiable risk factors. PAS is a prevalent obstetric emergency due to the rise in caesarean sections and placenta previa. Preventing maternal bleeding requires prompt and accurate PAS diagnosis. Before delivery, placenta accreta diagnosis allows collaborative planning in a specialized hospital to prevent mother and infant morbidity and mortality.
